# Optical design and commissioning results of a constant-imaging-distance fixed-included-angle grating monochromator at SSRF

**DOI:** 10.1107/S1600577525001778

**Published:** 2025-04-01

**Authors:** Lian Xue, Zhongliang Li, Junnan Liu, Huiting Chen, Gaofeng Zhao, Yong Wang, Wanqian Zhu

**Affiliations:** a Shanghai Advanced Research Institute Chinese Academy of Sciences Shanghai201210 People’s Republic of China; b Shanghai Synchrotron Radiation Facility, Shanghai201204, People’s Republic of China; University College London, United Kingdom

**Keywords:** beamline, fixed-included-angle grating monochromator, Shanghai Synchrotron Radiation Facility

## Abstract

A constant-imaging-distance fixed-included-angle grating monochromator has been designed and constructed at the Shanghai Synchrotron Radiation Facility to expand the covered energy range of the X-ray test beamline. The design and first commissioning results are presented in this paper. Initial results reveal the good performance of this monochromator, with a photon energy resolving power estimated to be over 8000 at the krypton *M*-edge for high line density gratings and a −1st order diffraction efficiency of the grating better than 30% for low line density gratings.

## Abstract 2

A constant-imaging-distance fixed-included-angle grating monochromator has been designed and constructed at the X-ray test beamline at Shanghai Synchrotron Radiation Facility to expand the covered energy range of the beamline.

## 1. Introduction

As the most important device in a beamline at a synchrotron radiation facility, a monochromator is used to transmit a mechanically selectable narrow band of wavelengths of radiation chosen from a wider range of wavelengths available at the input. Numerous types of grating monochromators have been designed and applied to different beamlines to meet various requirements. The variable-included-angle plane grating monochromator (VIA-PGM) (Petersen, 1982 ▸; Riemer & Torge, 1983 ▸; Pimpale *et al.*, 1991 ▸; Follath & Senf, 1997 ▸) is one of the most successful developed optics of the last few decades because of numerous advantages such as wide energy range coverage and flexible mode of operation. Based on these advantages, the VIA-PGM has been employed at many synchrotron radiation beamlines (Xue *et al.*, 2010 ▸; Aksela *et al.*, 1994 ▸; Follath, 2001 ▸; Warwick *et al.*, 2001 ▸; Follath *et al.*, 1998 ▸) all over the world.

At the Shanghai Synchrotron Radiation Facility (SSRF), to expand the covered energy range of the beamline, a new branch covering the soft X-ray energy range (80 eV to 1000 eV) has been designed and constructed on the X-ray test beamline (BL09B). BL09B is a bending magnet beamline used for at-wavelength measurements of beamline instruments and optics (Li *et al.*, 2019 ▸). The photon energy for the hard X-ray range is from 4 keV to 50 keV. Unfortunately, the VIA-PGM monochromator is unfavorable for the newly designed soft X-ray branch because a longer pre-mirror is required at the high energy end meaning that a larger space along the direction of beam propagation is needed to place the grating monochromator. However, as all of the equipment in the soft X-ray branch has to be added close to the existing hard X-ray branch in the same hutch, the space allowed for new equipment devices is very limited. In order to adopt a more compact design, the variable-included-angle grating monochromator was abandoned. The fixed-included-angle grating monochromator is an option for compact monochromators because off-axis rotation of the pre-mirror is not required during wavelength selection. The dragon-type monochromator is a typical choice of fixed-included-angle grating monochromators for reaching low photon energies (Yu *et al.*, 2001 ▸; Lai *et al.*, 2001 ▸), but its movable exit slit cannot always maintain a small spot size on samples. Therefore, it cannot fully meet the increased energy range of the soft X-ray branch of the test beamline.

A constant-imaging-distance fixed-included-angle grating monochromator (Hettrick, 1988 ▸; Hettrick *et al.*, 1988 ▸; Heimann *et al.*, 2011 ▸; Gerasimova *et al.*, 2022 ▸) has been designed and constructed for the X-ray test beamline at SSRF to expand the covered energy range of the beamline. In this work, the optical design and commissioning results of the monochromator are presented.

## 2. Optical schematic

**Figure 1:**
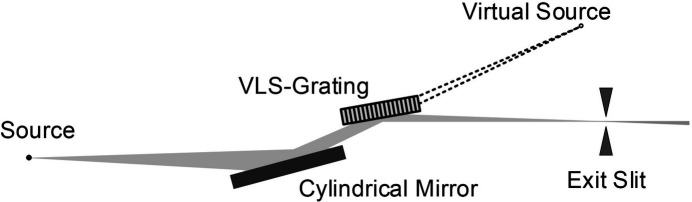
Optical schematic of the new optimized grating monochromator.

An optical schematic of the new optimized grating monochromator is shown in Fig. 1 ▸. In this design, a pre-focusing mirror is necessary to produce a converging beam and a virtual source behind the grating to meet the precondition*r*_1_ = −*r*_2_ where *r*_1_ is the objective distance and *r*_2_ is the imaging distance. Then, the grating produces a real image on the exit slit. The exit slit is the real focus of the pre-mirror.

The photon energy resolving power calculated in this study is mainly determined by seven factors: source size, exit slit size, meridian slope errors of the grating and focusing mirror, aberrations from the defocus and the coma, and the grating diffraction limit. Higher-order aberrations (smaller than *F*_30_) are small and negligible.

Two gratings and a cylindrical mirror are mounted in the grating monochromator. The parameters of the optical components are summarized in Table 1 ▸.

## 3. Commissioning results

### 3.1. Diffraction efficiency

**Figure 2:**
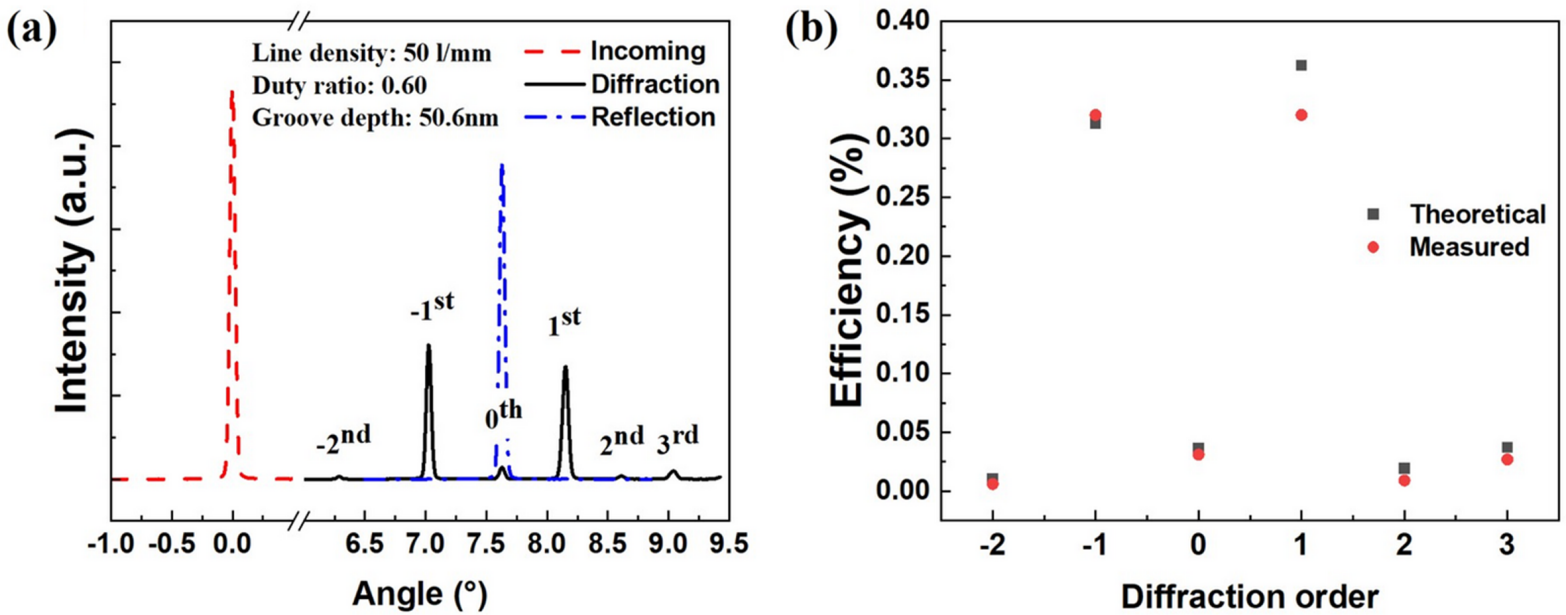
(*a*) Measured intensity of the incoming, diffracted and reflected beam of the 50 lines mm^−1^ grating. (*b*) Measured and theoretically predicted grating diffraction efficiency.

Comparing the two gratings, the 50 lines mm^−1^ grating aims to provide high photon flux, while the 350 lines mm^−1^ grating can provide relatively high energy resolving power. The combination of the two gratings can meet different experimental requirements. To achieve a higher diffraction efficiency, a duty ratio of 0.65 is optimized for both gratings, and the groove depth is chosen to be 50 nm for the 50 lines mm^−1^ grating and 12 nm for the 350 lines mm^−1^ grating. After preparation of the grating was completed, the basic parameters of the 50 lines mm^−1^ grating were characterized by atomic force microscopy. The measurement results show that the duty ratio of the grating is 0.60 and the groove depth is 50.6 nm. The grating was measured at the spectral radiation standard and metrology beamline (BL08B) at the National Synchrotron Radiation Laboratory (NSRL) to verify its actual diffraction efficiency, as shown in Fig. 2 ▸(*a*). The grating is fixed at a constant incident angle corresponding to a photon energy of 92.5 eV. A commercial photodiode (AXUV100G) located downstream of the grating was applied to measure the intensity of the incoming and diffracted/reflected beam by performing angular scanning in the direction of grating dispersion. The intensity of the incoming beam [dashed line in Fig. 2 ▸(*a*)] was measured by moving the grating out of the beam path, and the intensity of the reflected beam [dash-dotted line in Fig. 2 ▸(*a*)] was measured using the unruled margin area on the grating substrate to calibrate the incident angle of the grating. The measurement results show that the grating diffraction efficiency is basically consistent with the theoretical prediction as shown in Fig. 2 ▸(*b*), and the ±1st-order diffraction efficiency is greater than 30%.

### 3.2. Spot size

**Figure 3:**
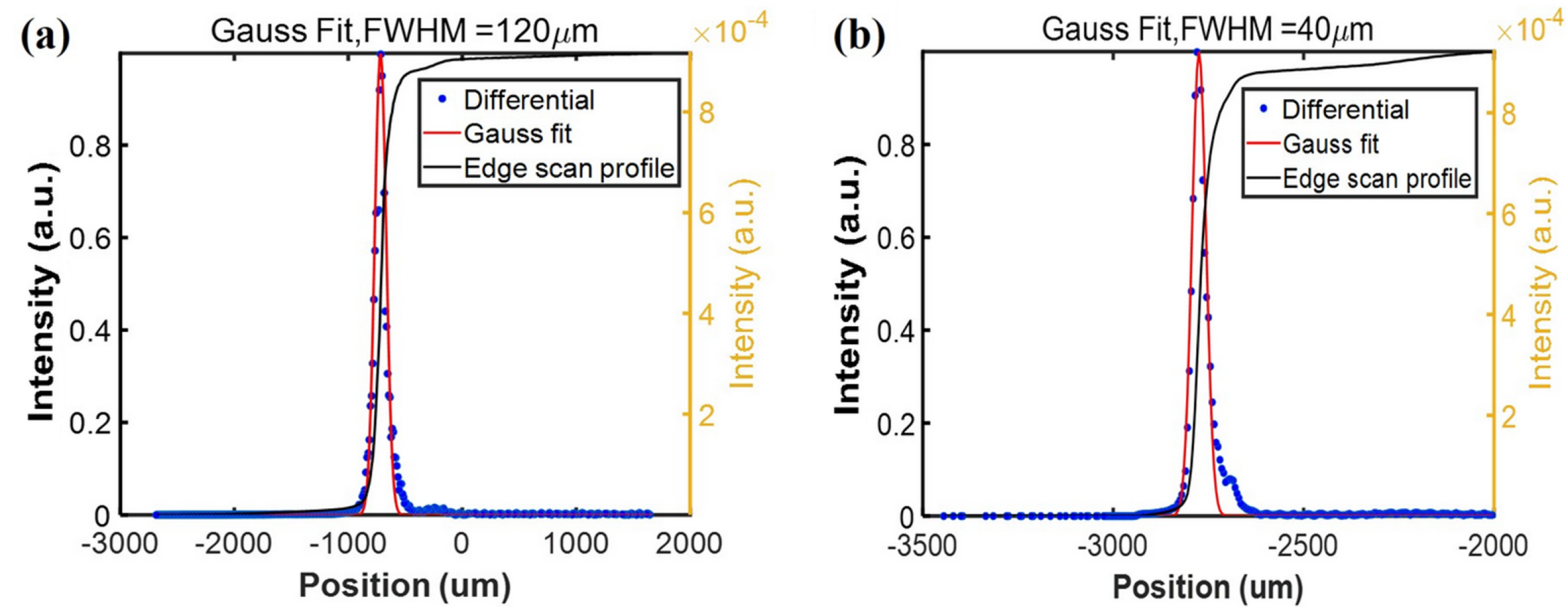
Measurement results of the zero-order diffraction of the grating obtained by edge scan.

Thanks to the independent movement of the four edges of the exit slit, the spot size at the exit slit could be measured using the edge scan method. The signal is recorded using a commercial photodiode behind the exit slit. Because the focus of the cylindrical pre-mirror is located at the exit slit, the focusing effect of the pre-mirror can be judged by measuring the zero-order diffraction of the grating. Fig. 3 ▸ shows the measurement results of the zero-order diffracted beam of the grating obtained by edge scan with a step of 5 µm in the vertical and 10 µm in the horizontal direction. In this measurement, the exit slit is fully opened in the other direction to guarantee that the photon beam is not obstructed. The differential results of the measurement curve show that the spot size of the zero-order diffracted beam of the grating is 120 µm in the horizontal and 40 µm in the vertical, which is consistent with theoretical predictions. This means that the grating monochromator is in a good focusing state, which can also be proven by the energy resolving power of the grating monochromator. For monochromatic beam, it is difficult to evaluate the vertical spot size at a certain wavelength since the grating is a linear dispersive element; whereas the horizontal spot size of the monochromatic beam is the same as for the white beam because the size of the focused spot is determined by an elliptical cylindrical focusing mirror located upstream of the monochromator.

### 3.3. Energy resolving power

The energy resolving power is a key parameter for evaluating a grating monochromator. A microchannel plate based ion chamber was designed and fabricated to evaluate quantitatively the energy resolving power of the beamline by measuring the excitation spectrum of a gas. The gas pressure in the ion chamber was 2 × 10^−6^ Torr to avoid the influence of gas collision broadening on the measurement results. The excitation spectra of different gases were measured to evaluate the energy resolving power of the monochromator.

**Figure 4:**
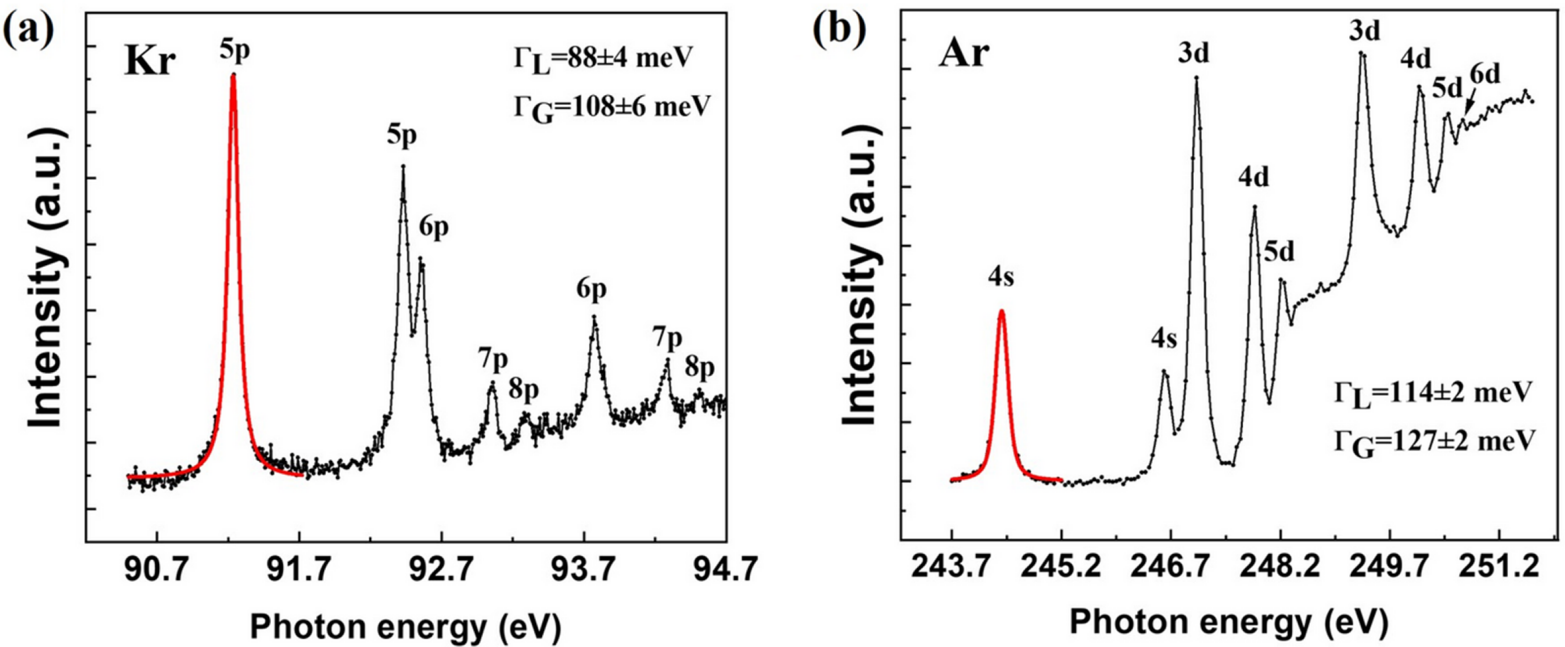
Excitation spectra of Kr and Ar gas measured using the 350 lines mm^−1^ groove density grating.

The Kr *M*_5_ (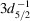) and *M*_4_ (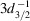) absorption edges were measured as shown in Fig. 4 ▸(*a*). The 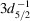 → 5*p* peak of Kr was fit using a Voigt profile, where the Lorentz width Γ_L_ is 88 ± 4 meV (Jurvansuu *et al.*, 2001 ▸) and the Gaussian width Γ_G_ is 108 ± 6 meV. Therefore, the resolving power can be readily calculated to be over 8400. The Ar *L*_3_ (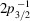) and *L*_2_ (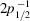) absorption edges were measured as shown in Fig. 4 ▸(*b*). The 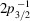 → 4*s* peak of argon was fit using a Voigt profile, where the Lorentz width Γ_L_ is 114 ± 2 meV (Kato *et al.*, 2007 ▸) and the Gaussian width Γ_G_ is 127 ± 2 meV. Therefore, the resolving power can be readily calculated to be over 1900.

**Figure 5:**
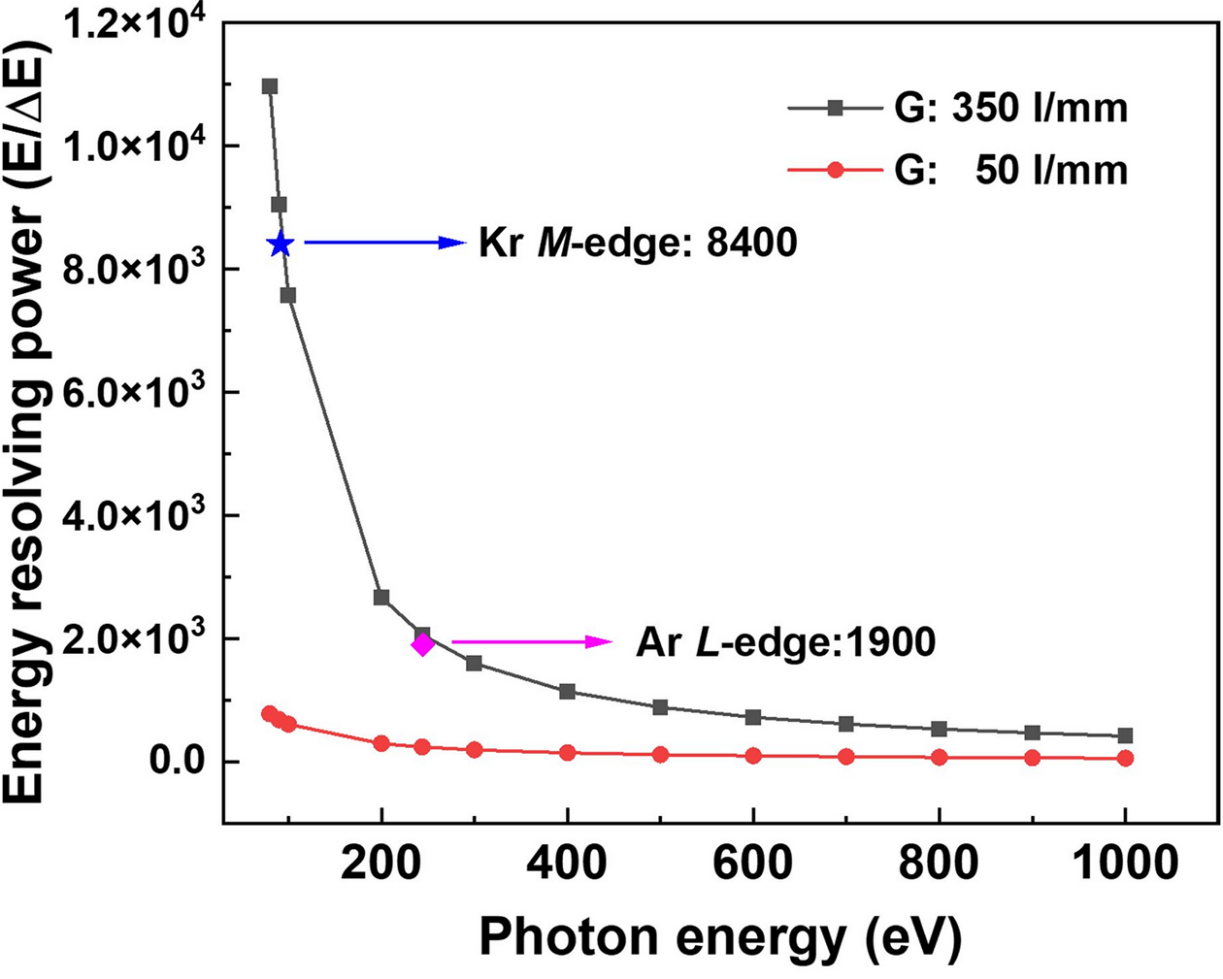
Theoretical curve and experimental data of the energy resolving power of the grating monochromator.

The theoretical energy resolving power of the grating monochromator with an exit slit size of 150 µm is shown in Fig. 5 ▸, where the slope error of both the grating and the pre-mirror is 0.2 µrad (r.m.s.) as shown in Table 1 ▸. The experimental data obtained from the measurements are in good agreement with the theoretically predicted values.

## 4. Summary

A constant-imaging-distance fixed-included-angle grating monochromator was designed and constructed for the X-ray test beamline at SSRF to expand the covered energy range of the beamline. Two gratings are employed in the monochromator to cover the energy range from 80 eV to 1000 eV. The core parameters of the 50 lines mm^−1^ grating were characterized and the measured results show that the −1st order diffraction efficiency of the grating was better than 30%. The grating monochromator has been installed on the beamline and is now under operation. Initial results reveal that the energy resolving power of the monochromator is estimated to be over 8000 at the krypton *M*-edge.

## Figures and Tables

**Table 1:** Specified parameters of the optical components in the grating monochromator VLS = variable line spacing.

	Pre-mirror	Grating 1	Grating 2
Surface shape	Cylindrical	Plane	Plane
Object distance (mm)	33755	−8509	−8509
Imaging distance (mm)	8755	8500	8500
Curvature radius (mm)	227768	–	–
Line density (lines mm^−1^)	–	50	350
VLS coefficient *b*_2_ (lines mm^−2^)	–	2.3 × 10^−4^	2.3 × 10^−4^
VLS coefficient *b*_3_ (lines mm^−3^)	–	1.387 × 10^−8^	1.384 × 10^−8^
Slope error (µrad, r.m.s.)	0.2	0.2	0.1
Coating material	Au	Au	Au
Optical area (mm)	200 × 40	180 × 30	180 × 30
Roughness (nm)	<0.3	<0.3	<0.3
